# Diminution of Oxidative Damage to Human Erythrocytes and Lymphocytes by Creatine: Possible Role of Creatine in Blood

**DOI:** 10.1371/journal.pone.0141975

**Published:** 2015-11-10

**Authors:** Neha Qasim, Riaz Mahmood

**Affiliations:** Department of Biochemistry, Faculty of Life Sciences, Aligarh Muslim University, Aligarh, Uttar Pradesh, India; Aligarh Muslim University, INDIA

## Abstract

Creatine (Cr) is naturally produced in the body and stored in muscles where it is involved in energy generation. It is widely used, especially by athletes, as a staple supplement for improving physical performance. Recent reports have shown that Cr displays antioxidant activity which could explain its beneficial cellular effects. We have evaluated the ability of Cr to protect human erythrocytes and lymphocytes against oxidative damage. Erythrocytes were challenged with model oxidants, 2, 2'-azobis(2-amidinopropane) dihydrochloride (AAPH) and hydrogen peroxide (H_2_O_2_) in the presence and absence of Cr. Incubation of erythrocytes with oxidant alone increased hemolysis, methemoglobin levels, lipid peroxidation and protein carbonyl content. This was accompanied by decrease in glutathione levels. Antioxidant enzymes and antioxidant power of the cell were compromised while the activity of membrane bound enzyme was lowered. This suggests induction of oxidative stress in erythrocytes by AAPH and H_2_O_2_. However, Cr protected the erythrocytes by ameliorating the AAPH and H_2_O_2_ induced changes in these parameters. This protective effect was confirmed by electron microscopic analysis which showed that oxidant-induced cell damage was attenuated by Cr. No cellular alterations were induced by Cr alone even at 20 mM, the highest concentration used. Creatinine, a by-product of Cr metabolism, was also shown to exert protective effects, although it was slightly less effective than Cr. Human lymphocytes were similarly treated with H_2_O_2_ in absence and presence of different concentrations of Cr. Lymphocytes incubated with oxidant alone had alterations in various biochemical and antioxidant parameters including decrease in cell viability and induction of DNA damage. The presence of Cr attenuated all these H_2_O_2_-induced changes in lymphocytes. Thus, Cr can function as a blood antioxidant, protecting cells from oxidative damage, genotoxicity and can potentially increase their lifespan.

## Introduction

Creatine (Cr) (1-methyl-guanidinoacetic acid) is a nitrogenous carboxylic acid that is endogenously produced in vertebrates or ingested from exogenous sources such as fish or meat. Dietary intake constitutes about 50% of total Cr content in humans and is 1–5 grams per day in an omnivore diet. Cr is synthesized in a two-step process from three amino acids: arginine, glycine and methionine. Cr synthesis begins in the kidney and pancreas from arginine and glycine. The guanidinoacetate produced is shuttled to the liver where it is methylated to give Cr which is then actively exported to tissues, such as muscles, where it is energetically required [1]. Almost 90–95% of the total Cr in human body is stored in the skeletal muscles which take up Cr against a concentration gradient with the aid of the sodium-dependent Cr transporter-1 [2]. Normally muscles maintain a high Cr concentration gradient which is 500–1000 fold higher inside the muscles than in plasma. In the muscles, 60% Cr is converted to phosphocreatine by Cr kinase using ATP (adenosine 5’-triphosphate) as the donor of phosphate group. Under conditions of acute energy demand, phosphocreatine transfers its phosphate group back to ADP (adenosine5’-diphosphate) quickly restoring ATP levels [3]. Thus, in muscles Cr represents the fundamental energy pool along with ATP.

These days dietary consumption of Cr supplements is on the rise. Cr is an efficient energy enhancer, improves physical performance, increases lean body mass and muscle endurance [4,5]. It can be safely taken in relatively high amounts (several grams per day) by normal healthy individuals without exhibiting any toxic effects [6]. Cr is, therefore, extremely popular as an ergogenic supplement in the sports industry and is widely used by athletes [7]. Even recreational exercisers and the elderly ingest Cr with the hope of improving physical activity. High oral dose Cr supplementation further elevates intracellular Cr and phosphocreatine pool in muscles and serum [8].

Apart from its use as an ergogenic aid, Cr supplementation is garnering more attention since it shows promising adjunct therapy in several pathological conditions [9]. Cr has been shown to be beneficial in the prevention and/or treatment of neurodegenerative disease (Huntington’s disease, Parkinson’s disease), amyotrophic lateral sclerosis, arthritis, muscular dystrophy, cardiovascular and metabolic disorders [10,11]. Cr enhances insulin sensitivity and increases glucose uptake in muscle cells [12]. Bender et al. [13] have reported that Cr supplementation resulted in a healthier and longer life span of mice.

Cr also displays antioxidant activity either in acellular or cellular systems. Cr was shown to be effective in direct scavenging of a range of radicals, including ABTS+ (2, 2'-azino-bis(3-ethylbenzothiazoline-6-sulphonic acid), superoxide anion and peroxy nitrite [14]. Later, Sestili et al. [15] showed that Cr also had protective cellular effect against three oxidants in several mammalian cell lines. Persons with Cr deficiency syndrome show increased oxidative stress and reactive oxygen species-induced apoptosis [16]. Recently, Cr was shown to protect rat kidney from damage by the anticancer agent cisplatin, which is known to exert nephrotoxicity by generation of free radicals [17]. However, there are several reports which either did not find any antioxidant property or any role for it, in the beneficial effects of Cr [18–20]. Thus, the antioxidant property of Cr remains a controversial issue and needs to be examined in more detail.

Cr is present in normal erythrocytes at 0.2 to 0.5 mM concentration [21]. Its concentration decreases with age of erythrocytes and has been used as an index of cell age and for estimating hemolytic processes [22]. Cr uptake in erythrocytes occurs by a sodium dependent mechanism located in the membrane [23] and its cellular concentration is ten times that in plasma [21, 24]. Unlike muscles and nervous tissue, where it has a key role in energy metabolism, there is no clear biological function for Cr in erythrocytes. In this study we have investigated the efficacy of Cr to protect human erythrocytes and lymphocytes from oxidative injury under in vitro conditions. Erythrocytes are highly susceptible to oxidative damage due to the high cellular concentration of transition metal ions, molecular oxygen and oxyhemoglobin, a potentially powerful promoter for the oxidative processes [25]. To extend our studies to nucleated blood cells, human lymphocytes were also treated with oxidant in the absence and presence of Cr. Lymphocytes are immune cells known as a source of both oxidant and pro-inflammatory compounds needed to support their functions. The ability to handle oxidative stress depends on mechanisms of protection either endogenously or provided by diet [26]. Therefore, it is important to maintain the antioxidant levels, redox homeostasis and, hence, the function of lymphocytes [27]. Our results show that Cr is an effective cytoprotectant and attenuates oxidant-induced damage to both erythrocytes and lymphocytes.

## Materials and Methods

### Chemicals

Hydrogen peroxide (H_2_O_2_), Cr, creatinine, tris(hydroxymethyl) amino- methane, S-acetylthiocholineiodide, thiobarbituric acid, HiSep 1077, trichloroacetic acid and 3-(4, 5-dimethylthiazol-2-yl)-2, 5-diphenyltetrazolium bromide (MTT) were purchased from Himedia Laboratories (Mumbai, India). Ouabain, 2, 2'-azobis(2-amidinopropane) dihydrochloride (AAPH), metaphosphoric acid, RPMI-1640 medium and 1-chloro-2, 4-dinitrobenzene were obtained from Sigma-Aldrich, USA. Reduced glutathione, 2, 4-dinitrophenylhydrazine and 5, 5’-dithiobisnitrobenzoic acid, ethidium bromide, agarose, Triton X-100 were from Sisco Research Laboratory (Mumbai, India). All other chemicals were of analytical grade.

### Isolation of erythrocytes from human blood

Young (22 to 30 years), healthy non-smoking volunteers were used as donors after getting their informed and written consent. The entire study, including methods used, was reviewed and specifically approved by the Institutional Ethics Committee (IEC) of Aligarh Muslim University that monitors research involving human subjects. Blood was collected in heparinised tubes and used immediately. It was centrifuged at 2000 rpm for 10 min at 4°C in a clinical centrifuge and the supernatant was removed. The erythrocyte pellet was washed three times with phosphate buffered saline (PBS) (0.9% NaCl in 10 mM sodium phosphate buffer, pH 7.4) and resuspended in PBS to give a 10% hematocrit.

### Treatment of erythrocytes, assay for hemolysis and preparation of lysates

Erythrocytes were treated with oxidant AAPH or H_2_O_2_ alone, Cr/ creatinine alone, and oxidant in presence of Cr/creatinine. Oxidative stress was induced by incubating 10% erythrocyte suspensions at 37°C with 50 mM AAPH for 4 h or 5 mM H_2_O_2_ for 2 h. In H_2_O_2_ treated erythrocytes, 1 mM sodium azide was added to inhibit catalase activity. In oxidant + Cr/ creatinine group, erythrocytes were first incubated with Cr/ creatinine (5, 10, 15 or 20 mM) for 30 min followed by addition of oxidant for the indicated period of time. In Cr/ creatinine alone group, erythrocytes were incubated with Cr/ creatinine at 37°C at 15 or 20 mM concentration. After these incubations, the erythrocyte suspensions were centrifuged at 2500 rpm for 10 min at 4°C. The supernatants were saved and their absorbance was recorded at 540 nm to determine the release of hemoglobin. Cell pellets were washed thrice with PBS and erythrocytes were lysed with ten volumes of hypotonic buffer (5 mM sodium phosphate buffer, pH 7.4) at 4°C for 2 h. The samples were centrifuged at 3,000 rpm for 10 min at 4°C and the supernatants (lysates) were quickly frozen in aliquots and used later for further analysis.

Hemoglobin in cell lysates was determined by cyanomethemoglobin method using Drabkins reagent (Hemocor-D Kit, Coral Clinical Systems, Goa, India). The oxidation of hemoglobin to methemoglobin was determined spectrophotometrically from the absorbance of hemolysates at 560, 576 and 630 nm [28].

### Isolation of human lymphocytes, treatment with H_2_O_2_ and preparation of cell lysates

Human blood was mixed with normal saline and Histopaque 1077 in 3:3:2 ratios. The mixture was centrifuged at 2400 rpm for 20 minutes and the white buffy middle layer consisting of lymphocytes was removed. The cells were pelleted by centrifugation at 2400 rpm, washed three times and resuspended in normal saline to give a 5% cell suspension which was used in further experiments.

Lymphocytes were incubated with H_2_O_2_ alone, Cr alone and oxidant in presence of Cr. Sodium azide (1 mM) was added to the cell suspension prior to incubation with H_2_O_2_ to inhibit catalase activity. Oxidative stress was induced by the addition of 5 mM H_2_O_2_ and incubation for 2 h at 37°C. In the Cr + H_2_O_2_ group, lymphocytes were first incubated with 10, 15 or 20 mM Cr for 30 min at 37°C followed by addition of 5 mM H_2_O_2_ and further 2 h incubation. In Cr alone group lymphocytes were incubated with 20 mM Cr at 37°C for 30 min. Control group received no treatments but cells were incubated at 37°C for 2 h. Following these treatments lymphocytes were microfuged at 12,000 rpm, the cell pellets were washed three times with normal saline and lymphocytes were lysed with 10 volumes of lysis buffer containing 0.5% Triton X-100, 100 mM NaCl, 1 mM EDTA and 20 mM Tris-HCl, pH 7.4 and incubation for 20 min at 4°C. Samples were again microfuged for 10 min at 12,000 rpm and the supernatants were quickly frozen in aliquots for further studies. Protein concentration in lysates was determined by the Lowry method using bovine serum albumin as the standard [29].

### Cell viability

Viability of isolated lymphocytes was checked by Trypan Blue exclusion test [30] and found to be > 93%.

MTT assay was done to study relative cell viability in the treatment groups. Stock solutions of MTT were prepared in PBS (5 mg/ml) and 10 μl added to 1 ml lymphocyte samples and incubated for 3 hours at 37°C. The samples were microfuged at 12,000 rpm for 5 min, the cell pellet was washed with PBS and then 1 ml dimethyl sulfoxide was added. The absorbance of blue formazan was determined at 570 nm [31].

### Malondialdehyde, carbonyl content and reduced glutathione

Malondialdehyde, a product of lipid peroxidation, was measured as thiobarbituric acid reactive substances [32]. The absorbance of pink chromophore produced during the reaction of thiobarbituric acid with malondialdehyde was measured at 535 nm. Carbonyl content in lysates was determined as an index of protein oxidation. Protein carbonyl groups react with 2, 4-dinitrophenylhydrazine to give an adduct whose absorbance was recorded at 360 nm [33]. The concentration of reduced glutathione was determined from its reaction with 5, 5’- dithiobisnitrobenzoic acid, after precipitation of proteins in cell lysates by metaphosphoric acid [34].

### Antioxidant enzymes and antioxidant power

The antioxidant status/power of cell lysates was determined from the ferric reducing antioxidant power (FRAP) [35] and 2, 2-diphenyl-1-picrylhydrazyl (DPPH) assays [36]. Antioxidant enzymes Cu, Zn superoxide dismutase and glutathione peroxidase were assayed from the inhibition of pyrogallol autooxidation and decrease in absorbance at 340 nm upon conversion of reduced nicotinamide dinucleotide phosphate to oxidized form, respectively [37,38].

### Assay of membrane bound enzymes

Na⁺, K⁺-ATPase (sodium potassium adenosine triphosphatase) activity was measured from the difference in inorganic phosphate released from ATP in the presence and absence of ouabain [39]. Acetylcholinesterase was assayed spectrophotometrically using S- acetylthiocholine iodide as the substrate in presence of 5, 5’-dithiobisnitrobenzoic acid. The absorbance of thio nitrobenzoate anion produced in the reaction was read at 410 nm [40].

### Scanning electron microscopy (SEM)

Erythrocytes (30 μl of 10% hematocrit) were fixed with 2.5% glutaraldehyde for 2 h and then centrifuged at 1500 rpm. The cell pellet was washed twice with PBS and resuspended in the same buffer. A drop of sample was spread on glass plates, air dried at 37°C and then washed with ethanol and distilled water [41]. The specimens were gold coated and finally observed under scanning electron microscope (SEM) at 1000x magnification.

### Comet assay

The single cell gel electrophoresis or comet assay was performed to assess DNA strand breaks in lymphocytes. Fully frosted glass slides were coated with 1% agarose and lymphocyte samples. After lysis, DNA was allowed to unwind for 30 min at 4°C in alkaline electrophoretic solution and subsequently electrophoresed for 20 min. Finally, the slides were neutralized, stained with ethidium bromide and analyzed under a CX41 fluorescence microscope (Olympus, Japan) [42]. The comets were scored at a magnification of 100x and images of 50 cells (25 from each replicate slide) for each sample were scored. Comet tail-length and olive tail moment were chosen as the parameter to assess nuclear DNA damage and were automatically generated by Komet 5.5 (USA) image analysis system.

### Statistical analysis

All data are expressed as mean ± standard error of mean (SEM). One way analysis of variance (ANOVA) was conducted to find out the significance of variations. A probability level of *p*<0.05 was selected, indicating statistical significance. All experiments were repeated five to six times to document reproducibility.

## Results

The antioxidant property of Cr was studied from its ability to protect cells from oxidative damage. Erythrocytes and lymphocytes are convenient models due to their ease of handling and availability. Two oxidants, AAPH and H_2_O_2_, were initially used that generate different types of free radicals. Several preliminary experiments were conducted on the basis of which erythrocytes were incubated with 50 mM AAPH for 4 h and 5 mM H_2_O_2_ for 2 h.

### Hemolysis

**Fig 1:**
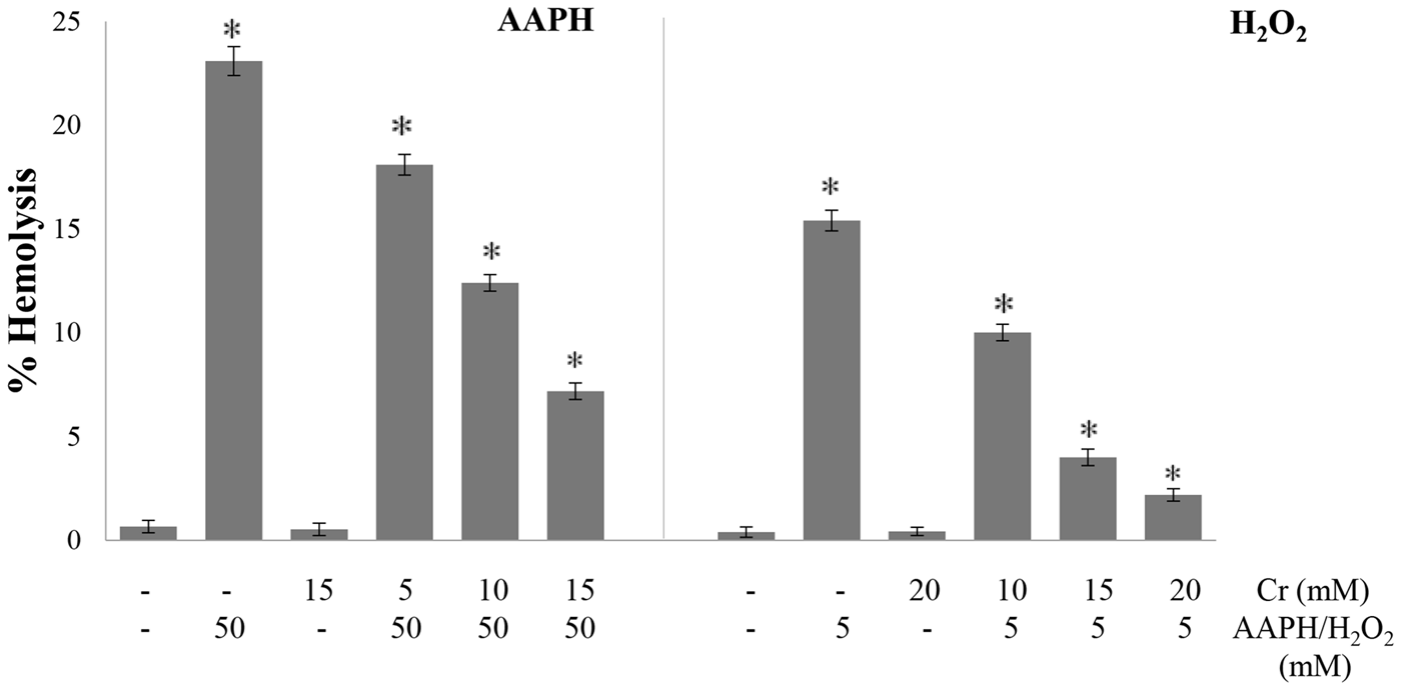
Effect of creatine on AAPH/ H_2_O_2_ -induced hemolysis of erythrocytes. Erythrocytes were treated with the indicated concentrations of H_2_O_2_ /AAPH, in presence and absence of Cr. After centrifugation, hemolysis was determined from the absorbance of supernatants as described in Materials and Methods. Results are mean values ± SEM from six independent experiments using blood from different donors. *Significantly different from control (p<0.05).

The ability of Cr to protect human erythrocytes from AAPH and H_2_O_2_ induced cell lysis was first studied. Cells were treated with oxidant for desired period of time and then centrifuged. Cell lysis was determined from the absorbance of supernatants at 540 nm due to hemoglobin released from damaged erythrocytes. Extensive hemolysis was induced in the presence of AAPH and H_2_O_2_ alone (Fig 1) while no lysis was observed in control cells incubated at 37°C for 4 h in the absence of oxidant and Cr. Erythrocytes pre-incubated with Cr and then challenged with the oxidants showed greatly reduced hemolysis; this protective effect of Cr was dose dependent. No hemolysis was seen when erythrocytes were incubated with 20 mM Cr showing that Cr, even at the high concentration used in this study, does not damage the cell.

### Malondialdehyde, carbonyl content and glutathione

**Fig 2:**
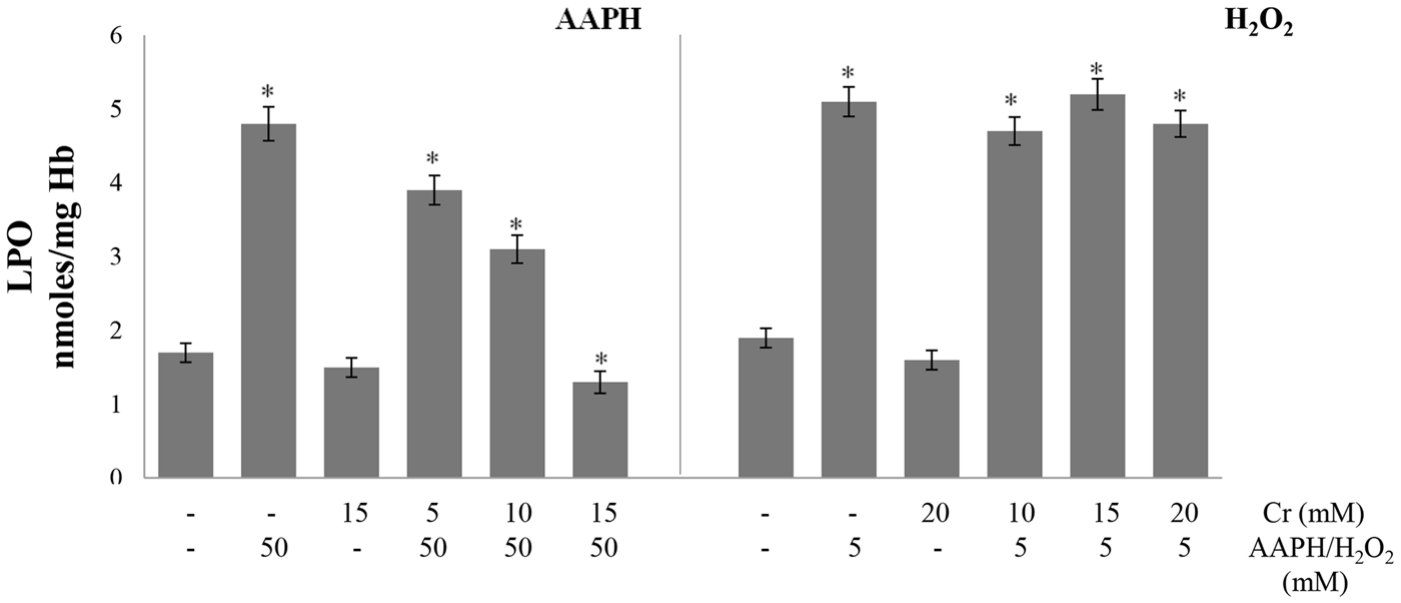
Effect of creatine on AAPH/ H_2_O_2_-induced lipid peroxidation. Erythrocytes were treated with the indicated concentrations of H_2_O_2_ /AAPH, in presence and absence of Cr. Malondialdehyde levels were determined in hemolysates as an index of lipid peroxidation. Results are mean values ± SEM from six independent experiments using blood from different donors. *Significantly different from control (p<0.05). LPO, lipid peroxidation.

**Fig 3:**
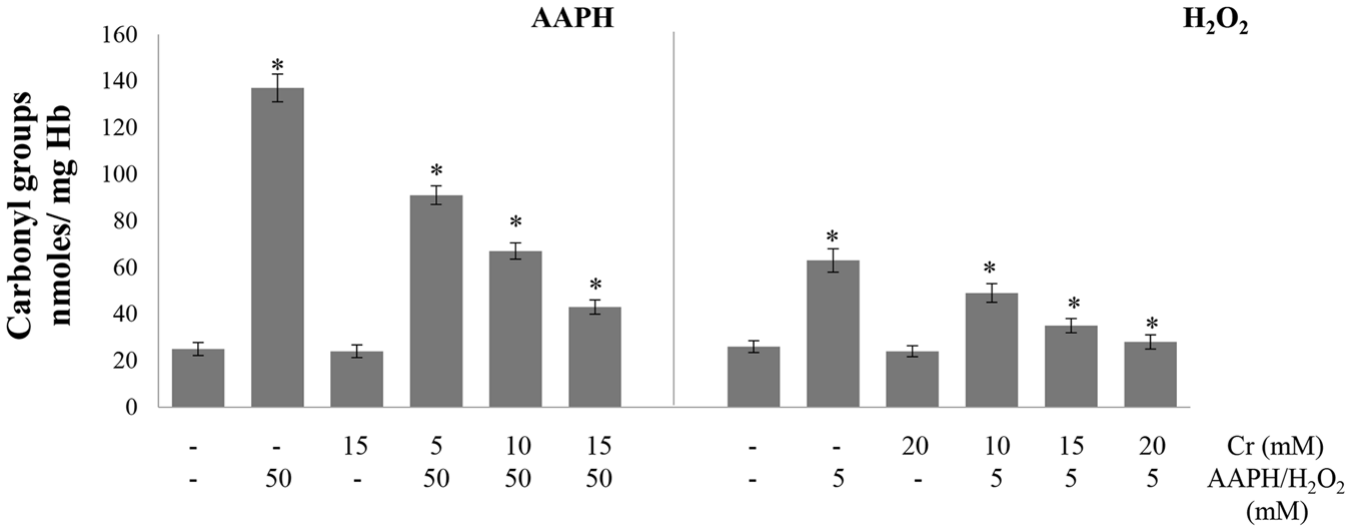
Effect of creatine on AAPH/ H_2_O_2_-induced protein oxidation. Erythrocytes were treated with the indicated concentrations of H_2_O_2_/AAPH, in presence and absence of Cr. Carbonyl content was determined in hemolysates as an index of protein oxidation. Results are mean values ± SEM from six independent experiments using blood from different donors. *Significantly different from control (p<0.05).

**Fig 4:**
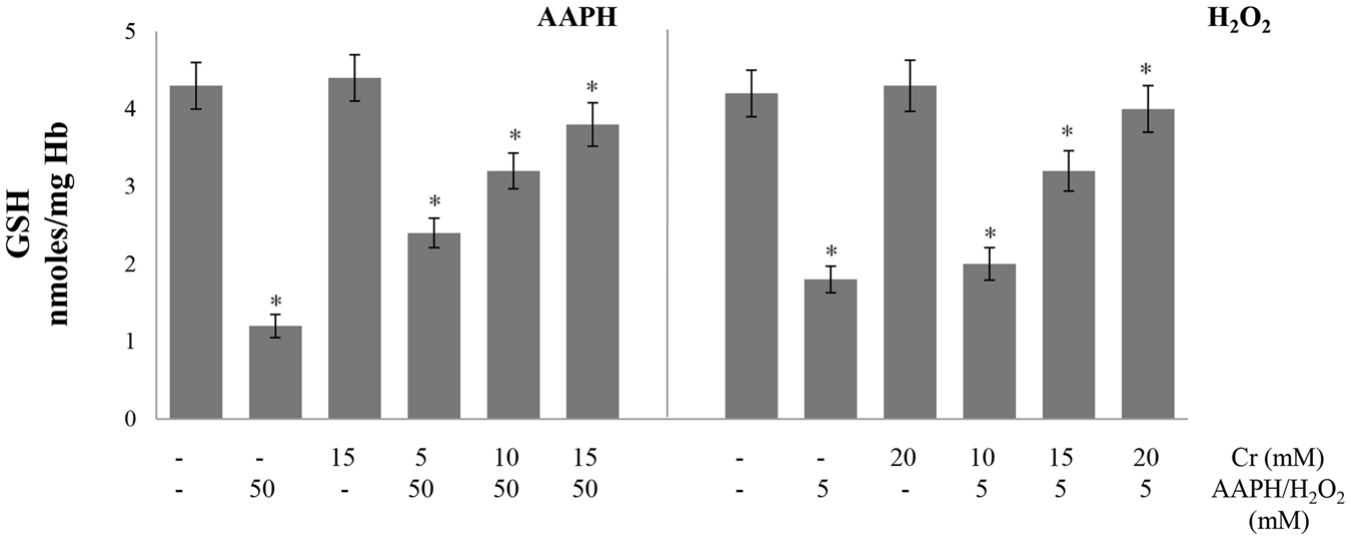
Effect of creatine on AAPH/ H_2_O_2_-induced changes in glutathione levels. Erythrocytes were treated with the indicated concentrations of H_2_O_2_/AAPH, in presence and absence of Cr. Reduced glutathione (GSH) levels were determined in hemolysates. Results are mean values ± SEM from six independent experiments using blood from different donors. *Significantly different from control (p<0.05).

Glutathione levels, lipid peroxidation and protein oxidation are commonly used as markers for the induction of oxidative stress in cell. The level of malondialdehyde, which is generated as an end product during the oxidation of lipids, was used as a marker of lipid peroxidation. Malondialdehyde reacts with thiobarbituric acid to form a pink product that absorbs at 535 nm. Oxidative stress also results in the generation of carbonyl groups in proteins which can be quantified from their reaction with 2, 4-dinitrophenylhydrazin resulting in the formation of a hydrazone adduct that absorbs at 360 nm. Treatment of erythrocytes with AAPH or H_2_O_2_ alone increased lipid peroxidation as shown by elevated malondialdehyde levels (Fig 2). Protein oxidation was also increased resulting in higher carbonyl content in hemolysates. This suggests the induction of oxidative stress in the cell. Pre-treatment of erythrocytes with Cr protected them from lipid peroxidation induced by AAPH but not by H_2_O_2_ (Fig 2). Protein oxidation, which was enhanced by both oxidants, was also greatly lowered in presence of Cr (Fig 3). Glutathione is a tripeptide which is the major non-enzymatic antioxidant of erythrocytes and is present in millimolar concentrations in the cell. Glutathione levels were lowered after incubation of erythrocytes with H_2_O_2_ or AAPH alone. However, a significant, recovery in glutathione content was seen in the presence of Cr. This AAPH and H_2_O_2_ induced reduction in glutathione levels again indicates the induction of oxidative stress in erythrocytes which was lowered by Cr (Fig 4).

### Antioxidant power and antioxidant enzymes

Superoxide dismutase is a potent antioxidant protective enzyme that scavenges superoxide anion by catalyzing its dismutation to H_2_O_2_ and oxygen while glutathione peroxidase catalyzes the conversion of H_2_O_2_ or organic peroxides to water and alcohol, respectively. The activity of both enzymes decreased after exposure of erythrocytes to H_2_O_2_ but was protected in the presence of Cr while Cr alone did not have any effect on enzyme activity (Table 1).

**Table 1:** Effect of creatine on H_2_O_2_-induced changes in methemoglobin levels, activity of erythrocyte membrane bound and antioxidant enzymes.

Treatments	MetHb	Na⁺, K⁺-ATPase	AChE	SOD	GP
**Control**	4.70±0.37	164.11±13.12	47.12±3.76	13.82±0.71	121.11±6.11
**5 mM H_2_O_2_**	30.14±2.41*	77.85±6.22*	16.79±1.34*	6.64±0.32*	48.34±2.41*
**20 mM Cr**	4.88±0.39	168.93±13.51	49.68±3.97	14.39±0.79	128.76±6.38
**10 mM Cr + 5 mM H_2_O_2_**	11.21±0.89*	104.56±8.36*	31.04±3.48*	8.63±0.43*	56.33±2.25*
**15 mM Cr +5 mM H_2_O_2_**	7.53±0.60*	126.49±10.11*	38.22±3.05*	10.91±0.55*	74.28±3.57*
**20 mM Cr + 5 mM H_2_O_2_**	5.28±0.42*	163.24±13.05*	44.50±5.56*	13.15±0.67*	83.97±4.73*

MetHb levels are in μmoles/l, Na⁺, K⁺ -ATPase activity is in nmoles/mg Hb/hr, AChE in nmoles/mg Hb/min, SOD in units/mg Hb/min and GP in nmoles/mg Hb/min Values are expressed as mean values ± SEM of six different experiments.

*Significantly different from control (*p*<0.05). MetHb; methemoglobin; Na⁺, K⁺-ATPase, sodium potassium adenosine triphosphatase; AChE, acetylcholinesterase; SOD, superoxide dismutase; GP, glutathione peroxidase; Hb, hemoglobin.

**Fig 5:**
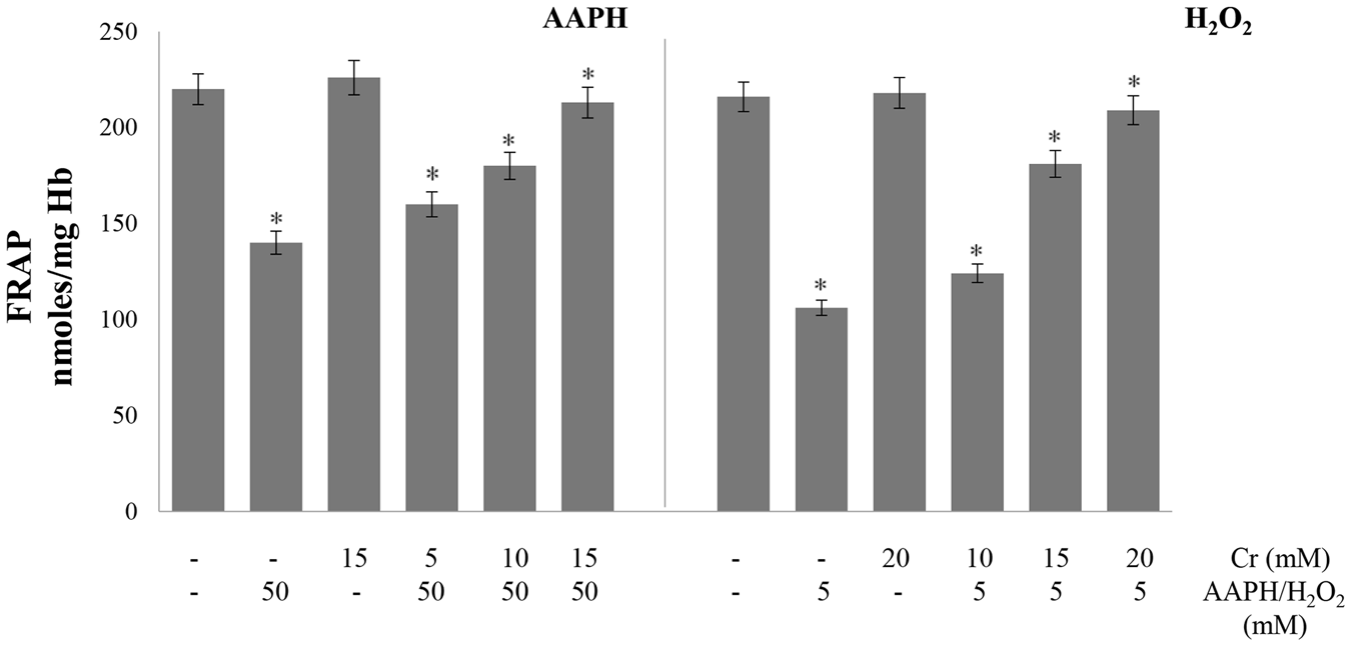
Effect of creatine on AAPH/ H_2_O_2_-induced changes in ferric reducing ability of cells. Erythrocytes were treated with the indicated concentrations of H_2_O_2_/AAPH, in presence and absence of Cr. FRAP assay was done in hemolysates. Results are mean values ± SEM from six independent experiments using blood from different donors. *Significantly different from control (p<0.05).

**Fig 6:**
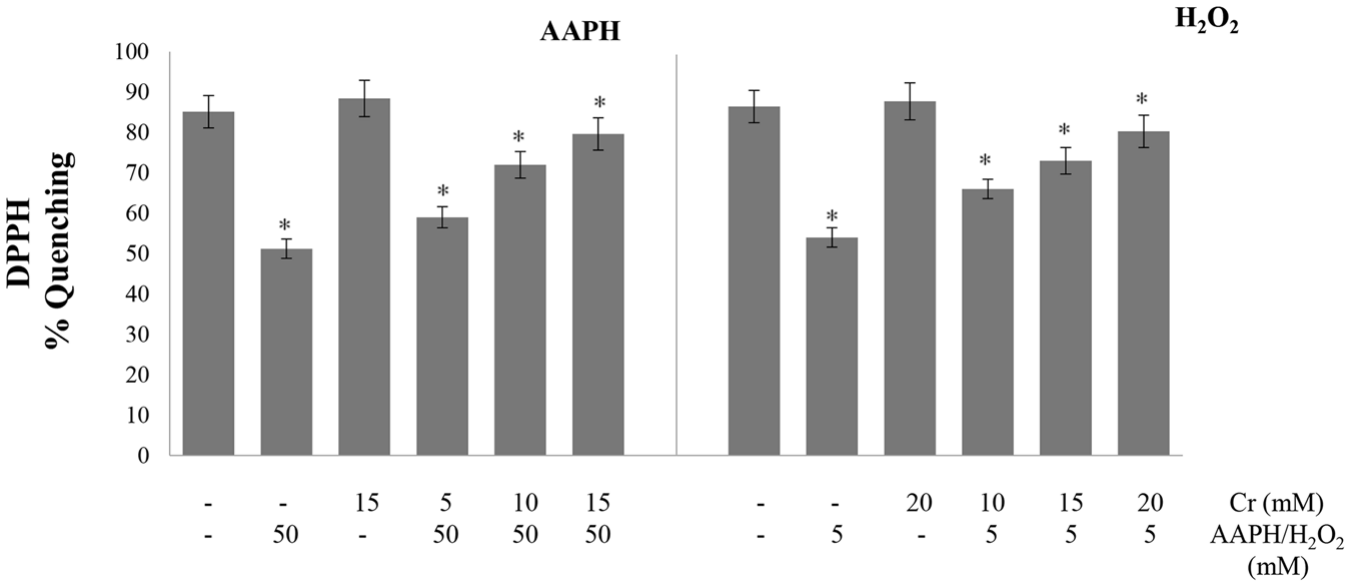
Effect of creatine on AAPH/ H_2_O_2_-induced changes in DPPH radical scavenging activity. Erythrocytes were treated with the indicated concentrations of H_2_O_2_/AAPH, in presence and absence of Cr. DPPH assay was done in hemolysates. Results are mean values ± SEM from six independent experiments using blood from different donors. *Significantly different from control (*p*<0.05).

Antioxidant power of the cell was determined from the ability to quench free radicals and reduce metal ions by employing the widely used DPPH and FRAP assays, respectively. The metal reducing activity was determined from the ability of hemolysates to reduce ferric ions to ferrous form using the FRAP assay. The treatment of erythrocytes with either oxidant alone lowered the ability of erythrocytes to reduce ferric ions but it was greatly restored by Cr (Fig 5). The DPPH radical scavenging activity of lysates was also lowered by treatment with oxidants alone but recovered when Cr was also present in the incubation medium (Fig 6).

### Methemoglobin formation

Another consequence of oxidative stress in the erythrocytes is the oxidation of ferrous ion of heme to the ferric form, converting hemoglobin to methemoglobin. The formation of methemoglobin is considered to be a marker of erythrocyte oxidative stress. H_2_O_2_ treatment increased methemoglobin levels which will decreases the oxygen carrying capacity of these specialized cells. These elevated methemoglobin levels were greatly lowered by Cr in a dose dependent manner (Table 1).

### Membrane bound enzymes

The membrane bound enzymes Na⁺, K⁺-ATPase and acetylcholinesterases were assayed to monitor membrane damage. The treatment of erythrocytes with H_2_O_2_ alone resulted in greatly reduced activity of both enzymes, compared to the control group (Table 1). This suggests membrane damage by the oxidant. However, presence of Cr in the reaction mixture resulted in recovery of both enzymes while Cr alone had no effect on the activity of either enzyme.

### Scanning electron microscopy

**Fig 7:**
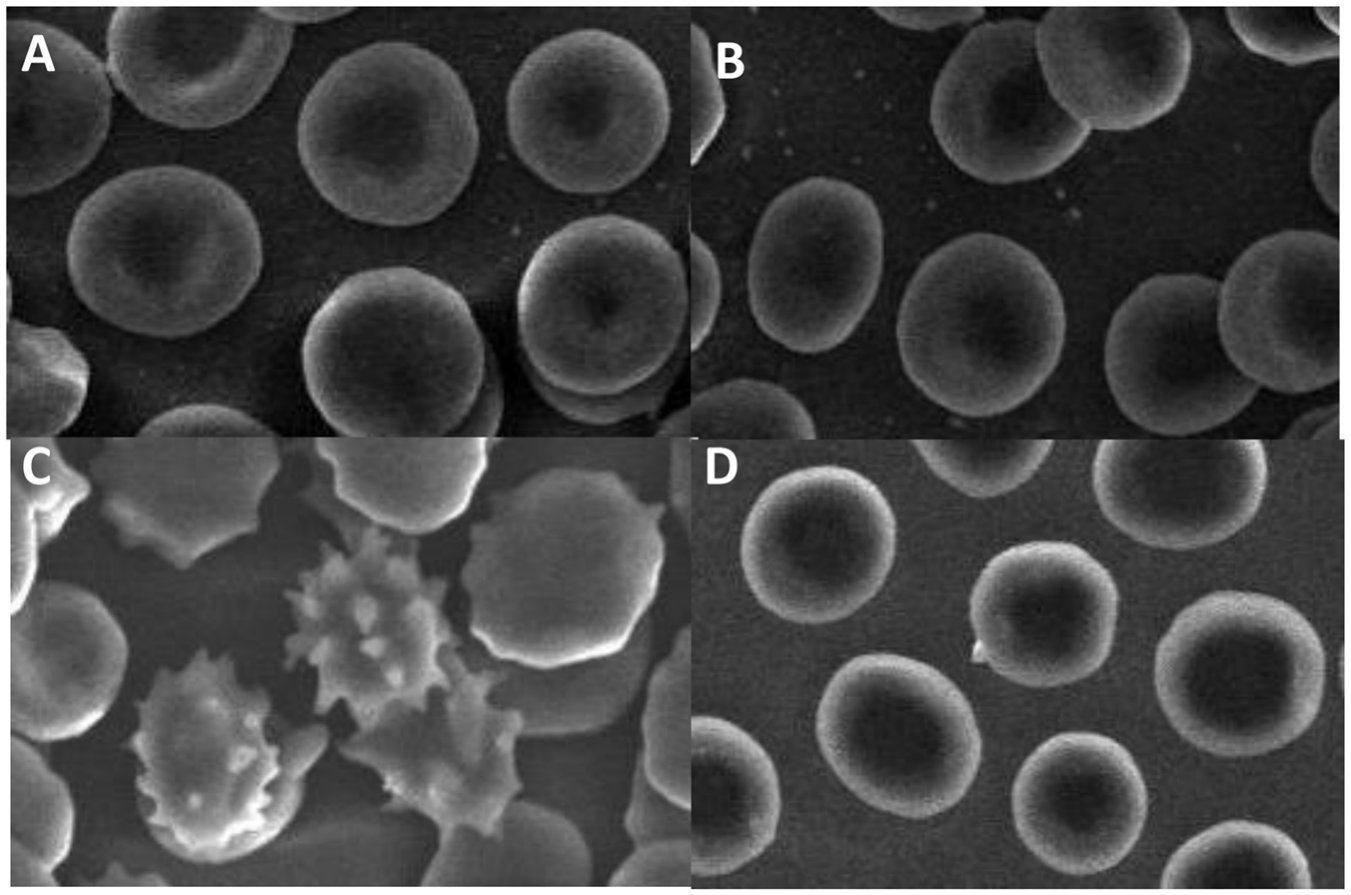
SEM images of human erythrocytes. (A) Untreated control; erythrocytes treated with (B) 20 mM Cr alone (C) 5 mM H_2_O_2_ alone and (D) 20 mM Cr + 5 mM H_2_O_2_. Treatment conditions are described in Materials and Methods section. Magnification is 1000 fold.

The SEM images of H_2_O_2_ treated erythrocytes showed distinct alterations in cell morphology. The cells had significant shape changes with finger like projections i.e. echinocytes, membrane disruption, cell blebbing, loss of biconcave shape (Fig 7). In some severely damaged cells, membrane rupture was also seen. Pre-treatment of erythrocytes with Cr protected cells from H_2_O_2_ induced damage. In Cr + H_2_O_2_ group the cell surface was smooth with homogeneous distribution of cells and biconcave shape showing that Cr effectively protects the cells from damage induced by H_2_O_2_. Importantly, 20 mM Cr alone did not alter the cell morphology which was similar to that of control untreated erythrocytes.

### Protection by creatinine

The efficacy of creatinine in protecting erythrocytes against AAPH/ H_2_O_2_ induced oxidative stress was studied next. Incubation of erythrocytes with 50 mM AAPH or 5 mM H_2_O_2_ increased erythrocyte hemolysis and carbonyl content, compromised antioxidant power and lowered the glutathione levels. Pre-treatment of cells with different concentrations of creatinine reduced these changes in a dose dependent manner. Creatinine itself did not damage the erythrocytes since the above mentioned parameters in the creatinine alone group were similar to control values (Tables 2 and 3).

**Table 2:** Effect of creatinine on AAPH-induced changes in some erythrocyte parameters.

Treatments	%Hemolysis	PO	GSH	FRAP	% DPPH quenching
**Control**	1.12± 0.08	22.48±1.79	3.97±0.31	217.87±17.42	84.51±6.76
**50 mM AAPH**	21.87±1.74*	133.72±10.69*	1.41±0.11*	147.19±11.77*	49.28±4.15*
**20 mM Crn**	0.92±0.07	24.13±1.93	4.16±0.33	224.42±19.35	85.77±6.94
**5 mM Crn + 50 mM AAPH**	17.68±1.41*	104.93±8.39*	2.10±0.16*	161.38±12.91*	53.61±4.28*
**10 mM Crn + 50 mM AAPH**	13.25±1.06*	67.15±5.37*	2.78±0.23*	173.20±13.89*	64.48±5.15*
**15 mM Crn + 50 mM AAPH**	9.80±0.78*	59.30±4.74*	3.02±0.24*	188.64±15.11*	69.15±5.53*

PO is in nmoles carbonyl groups per mg Hb, GSH levels are in nmoles/mg Hb and FRAP in nmoles/mgHb. Values are expressed as mean values ± SEM of six different experiments.

*Significantly different from control (*p*<0.05). Crn, creatinine; PO, protein oxidation; GSH, glutathione; FRAP, ferric reducing antioxidant power; DPPH, 2-diphenyl-1-picrylhydrazyl.

**Table 3:** Effect of creatinine on H_2_O_2_-induced changes in some erythrocyte parameters.

Treatments	%Hemolysis	PO	GSH	FRAP	% DPPH quenching
**Control**	0.94±0.07	25.33±2.12	4.11±0.56	213.62±17.08	85.63±6.85
**5 mM H_2_O_2_**	14.37±1.14*	61.04±4.88*	1.73±0.17*	112.21±9.97*	46.29±5.11*
**20 mM Crn**	0.81±0.06	26.95±2.15	4.32±0.41	221.37±19.69	86.61±6.70
**10 mM Crn + 5 mM H_2_O_2_**	13.98±1.11*	53.83±4.30*	2.31±0.32*	136.76±11.94*	52.18±4.17*
**15 mM Crn + 5 mM H_2_O_2_**	9.44±0.75*	47.74±5.81*	2.85±0.24*	157.34±13.58*	63.10±5.04*
**20 mM Crn + 5 mM H_2_O_2_**	5.65±0.45*	41.62±4.32*	3.19±0.13*	174.13±15.93*	72.44±5.79*

PO is in nmoles carbonyl groups per mg Hb, GSH levels are in nmoles/mg Hb and FRAP in nmoles/mgHb. Values are expressed as mean values ± SEM of six different experiments.

*Significantly different from control (*p*<0.05). Crn, creatinine; PO, protein oxidation; GSH, glutathione; FRAP, ferric reducing antioxidant power; DPPH, 2, 2-diphenyl-1-picrylhydrazyl.

#### Lymphocyte studies

We next examined the ability of creatine to protect human lymphocytes from H_2_O_2_-induced oxidative damage. Malondialdehyde, protein carbonyl content and other parameters in lymphocyte were used as markers of oxidative stress and cell damage. The levels of lymphocyte malondialdehyde in H_2_O_2_ alone group increased significantly as compared to control and Cr alone groups (Table 4). However, no statistically significant difference was observed between the malondialdehyde levels of 5 mM H_2_O_2_ alone and H_2_O_2_+ Cr groups even at high Cr concentration. The addition of 5 mM H_2_O_2_ alone increased protein carbonyls which were statistically more than those in control and Cr alone groups; pre-treatment of lymphocytes with Cr attenuated this increase. Similarly, the decrease in glutathione levels by H_2_O_2_ was protected by Cr in a dose dependent manner. The lymphocyte antioxidant power was greatly lowered by treatment with H_2_O_2_ alone but protected by Cr in a dose dependent manner. Lymphocyte membrane damage was analyzed from the activities of acetylcholinesterase and Na⁺, K⁺-ATPase. Pre-treatment of lymphocytes with Cr significantly protected both enzymes from inactivation by H_2_O_2_ (Table 4).

**Table 4:** Effect of creatine on H_2_O_2_-induced changes in some lymphocyte parameters.

Treatments	LPO	PO	GSH	FRAP	DPPH	Na⁺, K⁺- ATPase	AChE
**Control**	0.75±0.07	31.46±1.00	24.00±1.88	97.80±3.23	71.13±1.41	179.23±0.90	161.82±13.27
**5 mM H_2_O_2_**	1.14±0.10*	45.80±1.88*	13.63±1.20*	44.46±2.36*	44.73±2.85*	71.99±1.38*	115.03±11.27*
**20 mM Cr**	0.76±0.02	29.53±0.81	25.13±0.88	98.03±1.14	71.96±2.77	180.96±2.77	162.26±11.32
**10 mM Cr + 5mM H_2_O_2_**	0.99±0.05*	38.13±1.63*	19.33±0.38*	59.33±2.06*	55.86±1.45*	118.01±4.49*	134.73±11.31*
**15 mM Cr + 5mM H_2_O_2_**	1.05±0.05*	35.63±1.87*	21.50±1.76*	75.46±1.83*	62.60±1.04*	141.4±4.87*	146.80±12.80*
**20 mM Cr + 5mM H_2_O_2_**	0.94±0.01*	33.63±1.51*	23.31±1.84*	87.66±2.28*	67.60±1.20*	161.16±6.81*	156.83±12.44*

LPO, PO, GSH, FRAP are in nmoles/mg protein. Na⁺, K⁺ -ATPase activity is in nmoles/mg protein/hr and AChE in nmoles/mgprotein/min. Values are expressed as mean values ± standard deviation of five different experiments.

*Significantly different from control (*p*<0.05). LPO, lipid peroxidation; PO, protein oxidation; GSH, glutathione; FRAP, ferric reducing antioxidant power; DPPH, 2, 2-diphenyl-1-picrylhydrazyl, Na⁺, K⁺-ATPase, sodium potassium adenosine triphosphatase; AChE, acetylcholinesterase.

**Fig 8:**
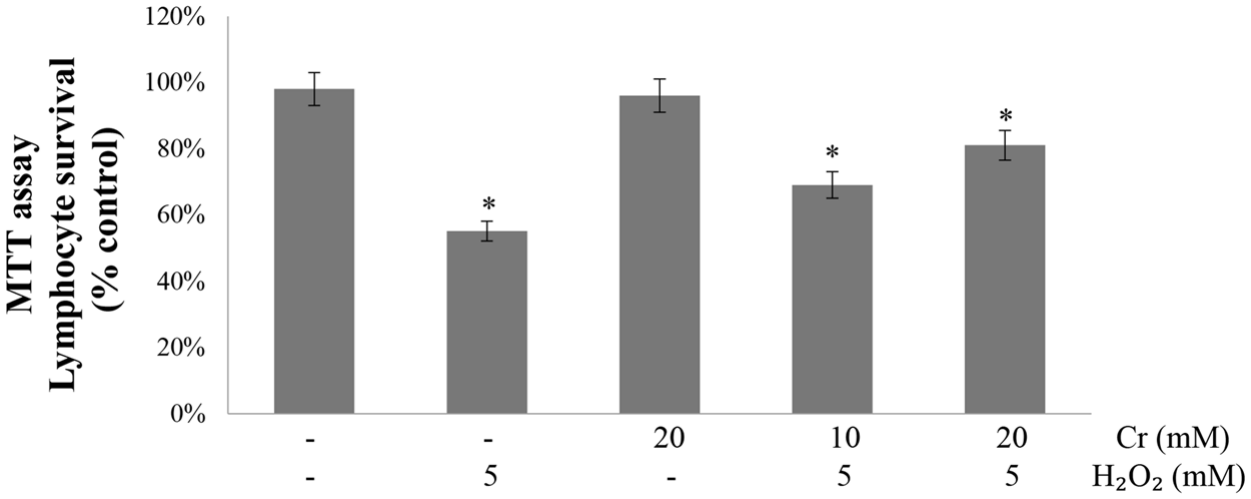
Effect of creatine on H_2_O_2_-induced decrease in lymphocyte viability. Human lymphocytes were treated with H_2_O_2_ in presence and absence of Cr and then their viability was determined by the MTT assay. Results are mean values ± SEM from five independent experiments using blood from different donors. *Significantly different from control (p<0.05).

The cell viability was determined from the MTT assay. MTT (3-(4, 5 dimethythiazol-2-yl) -2, 5-diphenyl tetrazolium bromide) is reduced to an insoluble blue formazan product by the mitochondrial enzyme succinate dehydrogenase [31]. Therefore, the amount of formazan produced is directly proportional to the number of living cells. As expected, 5 mM H_2_O_2_ significantly decreased lymphocyte viability as compared to control samples. The Cr treatments partially reverted the cytotoxicity caused by H_2_O_2_; 20 mM Cr provided good protection against H_2_O_2 –_induced cell killing (Fig 8).

**Fig 9:**
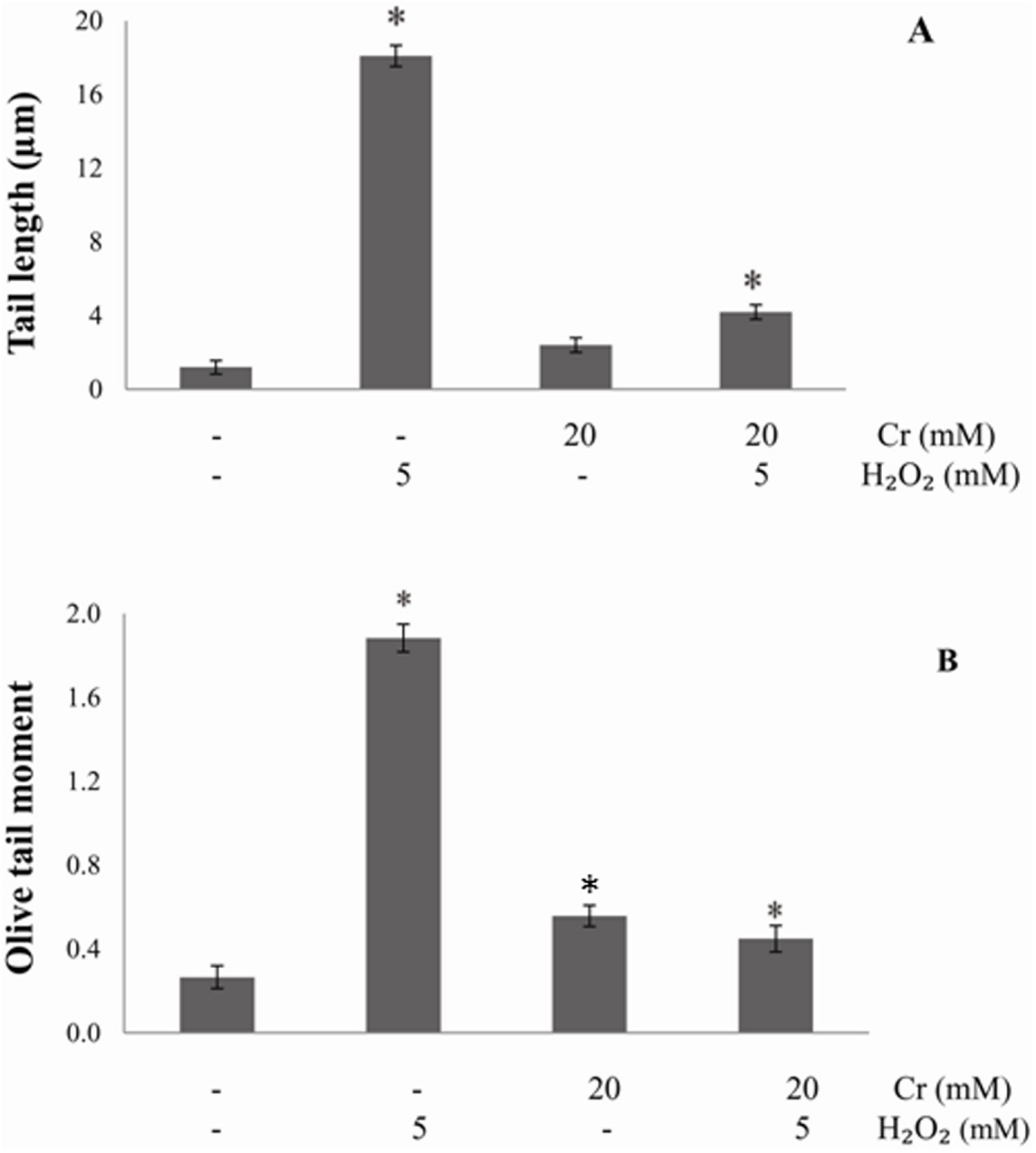
Analysis of DNA damage by comet assay. DNA damage in human lymphocytes after incubation with H_2_O_2_, in presence and absence of Cr, was studied by the Comet assay as described in Materials and Methods. (A) Tail length (B) Olive tail moment. Results are mean values ± SEM from five independent experiments using blood from different donors. *Significantly different from control (p<0.05).

The genotoxic effects of H_2_O_2_ and the protective ability of Cr were examined in lymphocytes using the comet assay. Due to DNA damage or fragmentation, higher DNA percentage in tail region was observed in H_2_O_2_ groups as evidenced by increase in comet tail length and olive tail moment. This indicates increased level of DNA single strand breaks and alkali labile sites in the oxidant treated cells. Pre-treatment of the cells with Cr significantly reduced the genotoxicity of H_2_O_2_ and there was a decrease in comet tail length and olive tail moment (Fig 9).

## Discussion

Creatine is widely used in the sports industry to improve athletic performance. The beneficial effects of Cr supplementation are also recommended in a wide spectrum of diseases, many of which involve increased generation of free radicals and reactive oxygen species. Recent studies have shown that the antioxidant property of Cr may play a beneficial role in its action by reducing endogenous production of reactive oxygen species. However, the extent to which the antioxidant property of Cr contributes to its cytoprotective effects has not been well studied and remains a point of controversy. One way to address this is to challenge cells with different oxidants and then determine their response. In the present study we have examined the role of Cr in protecting human blood cells from oxidant-induced injury. Erythrocytes and lymphocytes were selected to represent effect on non-nucleated and nucleated blood cells.

Erythrocytes are the most abundant cells in blood and contain Cr whose function in these cells is unknown. Erythrocytes lack DNA, organelles and protein synthesizing machinery and, therefore, represent a simple and convenient cellular model system to study toxicant-induced damage. Two different free radical inducing compounds, H_2_O_2_ and AAPH, were selected to check the versatility of Cr as an antioxidant. AAPH decomposes to give nitrogen and initiating peroxyl radical [43] whereas H_2_O_2_ is a non-radical reactive oxygen species that is membrane diffusible and can react with redox active metals like iron to generate the damaging hydroxyl radical. Erythrocytes were challenged with H_2_O_2_/AAPH, in the presence and absence of Cr. Several parameters were then determined to see if Cr could mitigate oxidant-induced oxidative stress and cell injury in erythrocytes.

Treatment of erythrocytes with AAPH and H_2_O_2_ resulted in cell damage with 20–30% hemolysis but this was prevented in a dose dependent manner by pre-treatment of cells with Cr. Incubation of erythrocytes with either oxidant also resulted in increase in lipid peroxidation. Peroxyl radicals generated by AAPH oxidize lipids that cause loss of membrane integrity. Malondialdehyde, one of the end products of lipid peroxidation, can also cross-link proteins and phospholipids resulting in membrane dysfunction and diminished cell survival [25]. AAPH-induced lipid peroxidation was decreased in Cr pre-treated cells indicating that Cr can protect membrane lipids from oxidation. However, Cr was ineffective in reducing H_2_O_2_ induced lipid peroxidation in erythrocytes in repeated experiments. Similar results were obtained by other workers also. Lawler et al. [14] found that Cr did not protect against t-butyl hydroperoxide induced lipid peroxidation in an acellular setting. Cr supplementation also did not reduce exercise induced lipid peroxidation in liver and muscles of rats [44,45]. Since Cr protects against H_2_O_2_ induced hemolysis, but not lipid peroxidation, this indicates that lipid peroxidation does not play a major role in H_2_O_2_ induced cell damage and lysis. Further, the protective effect of Cr on AAPH, but not H_2_O_2_, induced lipid peroxidation shows that the mechanism/radical involved in the peroxidative effects of these two oxidizing agents is different in erythrocytes.

Oxidative damage often leads to loss in protein function due to their oxidation that introduces carbonyl groups [46]. The accumulation of protein carbonyls results from oxidative damage to the protein backbone, direct oxidation of amino acid side chains or binding of aldehydes produced from lipid peroxidation [47]. Both AAPH and H_2_O_2_ increased carbonyl content indicating elevated protein oxidation in the cell. Cr was able to shield erythrocytes from modification and prevented build up of carbonyl groups. This is the first example where Cr has been shown to protect against oxidative modification of proteins. This is important since oxidatively damaged proteins, despite having long half life, are not repaired and are ultimately removed by proteolytic degradation.

Cr also attenuated the depletion in glutathione level by H_2_O_2_ and AAPH. Glutathione works as a co-factor for various metabolic enzymes, helps in intracellular transport, functions as an antioxidant, facilitates protein folding and degradation, and maintains membrane protein thiol groups in reduced form [48]. The decline in glutathione content causes an imbalance in the redox system of the cell [48] that will compromise the ability of erythrocytes to cope with a generalized increase in oxidative stress. The induction of oxidative stress and depletion of glutathione will, in turn, lower the antioxidant power of cell. This was studied by employing two widely used assays. The free radical (DPPH.) scavenging and metal (Fe_3+_) reducing activities, determined by DPPH and FRAP assays, were impaired upon treatment of cells with AAPH and H_2_O_2_. However, it was restored in a dose dependent manner by pre-loading erythrocytes with Cr. This boost in the antioxidant capacity is direct evidence that oxidative stress declined when cells were treated with Cr.

Further protective experiments employed only H_2_O_2_. Membrane damage, suggested by hemolysis experiments, was also monitored by activity of marker enzymes, acetylcholinesterase and Na⁺, K⁺-ATPase. Loss of membrane integrity is monitored by the inhibition of Na⁺, K⁺-ATPase, which maintains Na⁺ and K⁺ gradient across plasma membrane that is important for cell function [49]. Studies have shown a correlation between acetylcholinesterase inhibition in blood and damage in target tissues [50]. Na⁺, K⁺-ATPase and acetylcholinesterase activities declined in erythrocytes in presence of H_2_O_2_ but Cr pre-treatment greatly restored them towards control levels. Cr also prevented the H_2_O_2_-induced inactivation of antioxidant enzymes.

Hemoglobin is a major target of reactive oxygen species in erythrocytes due to its abundance. One consequence is the conversion of ferrous iron of hemoglobin to ferric form giving methemoglobin which is inactive as an oxygen transporter. Methemoglobin formation, in fact, is considered to be a marker of erythrocyte oxidative stress [25]. H_2_O_2_ induced high stress condition in erythrocytes while Cr stabilized the ferrous form of heme iron and prevented methemoglobin formation.

The scanning electron microscopy results strongly support our biochemical studies. The electron microscopy images show that H_2_O_2_ caused morphological changes in the erythrocytes, from discoid to an echinocytic form. These structural changes can be due to insertion of foreign molecules in either the outer or inner monolayer of erythrocyte membrane [51]. Cr (at 20 mM) was very effective in protecting erythrocytes from oxidant-induced damage and greatly restored the erythrocyte cell morphology. The cell surface was smooth without any protrusions and the shape looked very much like that of control cells.

Lymphocytes are the cells of immune system involved in defending the body against infectious diseases and foreign materials. Unlike erythrocytes, lymphocytes are complete cells because they contain nucleus and other cellular organelles. The number of white blood cells in the blood is often an indicator of disease. Lymphocytes were used to assess the role of Cr as antioxidant in protecting nucleated cells from oxidant-induced damage. Our results show that Cr protected lymphocytes also from oxidant induced damage as monitored from the levels of protein carbonyls and glutathione levels. Here too inclusion of Cr to the samples did not lower lipid peroxidation, confirming previous observations [14,44,45] and our present results on erythrocytes. Cr also protected lymphocyte cell membrane and prevented Na⁺, K⁺-ATPase and acetylcholinesterase from H_2_O_2_ induced inactivation. Increase in Na⁺, K⁺-ATPase of the lymphocyte membrane increases Na⁺ in the cell that would lead to a significant increase in pump activity [52]. Changes in lymphocyte acetylcholinesterase activity reflect immune deficiency related to cell dysfunction [53]. Importantly, the H_2_O_2_ induced decrease in cell viability was also attenuated by Cr. Cr modulated H_2_O_2_ induced DNA damage in human lymphocytes. The protective genotoxic effect of Cr was assessed by comet assay, where an increase in tail length indicates DNA impairment. The amount of DNA that migrates in the tail, thus making the shape of a comet, due to treatment with a toxicant is correlated with DNA damage [54]. Hydrogen peroxide is believed to cause DNA strand breakage by generating hydroxyl radical close to the DNA molecule, via the Fenton reaction resulting in DNA instability, mutagenesis and ultimately carcinogenesis [54]. Lymphocytes incubated with H_2_O_2_ alone showed greater DNA strand breakage in contrast to untreated controls or cells incubated with Cr alone. Cr at 20 mM was very effective in inhibiting DNA strand breakage by H_2_O_2_. These results provide evidence that Cr can function as an antioxidant in the nucleated human lymphocytes as well and protects cellular organelles also from the damaging effects of oxidative stress condition. In near future, it will be necessary to investigate pathways being activated in lymphocytes after the treatment with Cr.

The ergogenic effects of Cr supplementation have been primarily attributed to the role of Cr in the energy system in muscles. The demonstration by Lawler et al. [14] that Cr exhibits antioxidant property opened up the possibility that at least some of its beneficial effects in athletes may be due to lowering of the exercise induced reactive oxygen species and oxidative stress [55,56]. Our results suggest that Cr supplementation can also be beneficial by (i) reducing the buildup of oxidants, not only in muscles but also in blood, and (ii) increasing the oxygen carrying capacity of blood by inhibiting conversion of hemoglobin to methemoglobin, which is inactive as an oxygen carrier. Following Cr loading, the increase in its concentration is relatively greater in erythrocytes than in other tissues [57]. The protection against oxidative injury by Cr may also increase cell life time since damaged erythrocytes are removed from circulation by spleen. This will result in less stress on red bone marrow to produce replacement erythrocytes. There is, infact, a direct co-relation between Cr levels and erythrocyte survival time in hemolytic patients [24]. Cr content in young cells is 6–9 fold greater than in older ones [58] and erythrocyte Cr levels have long been used as an index of cell age [59]. Since reactive oxygen species are thought to regulate erythrocyte lifespan in blood [60], low Cr concentration will render the old erythrocytes more vulnerable to oxidative damage leading to their removal from circulation.

The protective effects of Cr in erythrocytes and lymphocytes can be attributed to its direct antioxidant activity by quenching oxidant-induced free radicals and reactive oxygen species and/or enhancing the cellular enzymatic and non-enzymatic antioxidant defence system e.g. attenuating oxidant-induced decrease in glutathione levels and inactivation of antioxidant enzymes. The Cr effects cannot be mediated by its impact on cellular energy metabolism since erythrocytes and lymphocytes lack Cr kinase and cannot convert Cr to phosphocreatine. Reduction of oxidative stress by chelation of iron is also not likely since Cr, unlike many antioxidants like plant polyphenolic compounds, does not bind iron [15]. However, the exact mechanism by which Cr exerts its antioxidant activity remains to be elucidated and should be the subject of future studies.

Creatinine, a by-product of Cr and phosphocreatine metabolism, was also analysed for antioxidant properties. Creatinine is present in plasma and RBC and exhibits promising results by decreasing oxidative damage induced by AAPH and H_2_O_2_, almost to the same extent as Cr. There has been, surprisingly, no study examining the antioxidant property of creatinine and our results represent the first report on the potential antioxidant function of this compound. Since creatinine has a structure very similar to Cr, its protective effect can also be explained on the basis of antioxidant properties.

Although the concentrations of Cr used in this study are high, similar concentrations were used previously by other workers [14,15,61]. They attribute to the fact that Cr is a weak antioxidant compared to other well known antioxidans like plant polyphenols, vitamins C and E, Trolox, glutathione etc. that are effective at much lower (submillimolar) concentrations and against a wider array of free radicals/ reactive oxygen species. However, it should also be remembered that Cr supplements are often taken in large amounts (20–30 gram per day for several days) and result in elevated plasma Cr levels that can reach 2–3 mM [8] while Cr concentration in muscles is in the range of 20–40 mM [62]. In addition, as mentioned by Sestili et al. [63], Cr is a ubiquitous “physiologically on-board” molecule and thus its effects will be enhanced due to its long term association with cellular components. The beneficial effects of Cr may, therefore, be better than that of many plant antioxidants which may greatly improve its effectiveness and efficacy in the cell.

The role of Cr as an antioxidant in blood and its ability to minimize hemolysis and methemoglobin formation can have important clinical manifestations also. In oxidatively damaged erythrocytes with damaged or ruptured cell membrane, the generated reactive oxygen species escape the cells and have tremendous potential to damage other components of the circulation [64]. Most importantly there is reduction in the capacity to load and unload oxygen. Anemic patient suffers from tissue hypoxia, the consequence of a low oxygen-carrying capacity of the blood [65] usually due to formation of methemoglobin. Insufficient supply of oxygen and nutrients can also trigger events like viral infections and bacterial infections. So the antioxidant function of blood Cr maybe of paramount importance to keep oxidative damage in these cells to a minimal level.

The flow of blood and the flow of erythrocytes in particular within the circulation depends on the characteristic ability of erythrocytes to undergo deformation when subjected to a shear stress [66]. Echinocyte is a form of red blood cell with an abnormal cell membrane with spaced thorny projections, found in patients with uremia, pyruvate kinase deficiency, hypomagnesemia, hypophosphatemia, and hemolytic anemia in long-distance runners [67]. It is likely that the echinocytosis is the cause of the hemolytic anaemia as well [68]. These cells were also shown to develop in vivo during hemodialysis, and disappear at the end of the procedure; increase in blood viscosity is often seen during this procedure [69] which may affect the circulation in patients [70]. Erythrocyte morphology is related to the pathophysiology of alcoholic liver disease and abnormal erythrocytes are observed in such situations [71]. We suppose that including Cr in diet can avoid the eruptions of echinocytes and help in smooth, unrestricted blood flow.

The property of Cr to protect DNA from oxidative damage might be important in diseases like cancer. Humans are constantly exposed to natural DNA damaging agents such as sunlight, dietary agents like cooked meat, acrylamide etc. and endogenously formed oxygen free radicals. Induction of DNA damage is thus a fundamental and unavoidable process that plays a key role in cancer development and the induction of heritable genetic defects [72]. These accumulated DNA mutations are the cause for the decline in gene expression and loss of functional capacity seen with increasing age and may be mitigated by Cr.

Cr may also have a potential function in disorders where oxidative stress plays a key role in the pathogenesis of the disease and where it is increased in blood [73]. Oxidative injury is increased in patients with diabetes mellitus because of a weakened defense due to reduced endogenous antioxidants such as reduced glutathione and vitamin E [74]. It is believed that oxidative insult is the pathogenic mechanism leading from insulin resistance to overt diabetes. The ability of Cr to prevent or reverse oxidant stress may be important for its clinical usefulness [12,74] and, thus, Cr supplementation may be beneficial in patients with diabetes. In hemolytic anemia there is intravascular hemolysis which releases erythrocyte contents into the blood stream. This largely includes iron-containing compounds capable of doing damage through Fenton reactions [75], which can be minimized by Cr supplementation. Other disorders with increased oxidative stress in blood, and where Cr supplementation may be useful, are neurodegenerative diseases like Alzheimers and cardiovascular diseases [13, 76–78].

Increased K⁺ loss is common to several red blood cell disorders suggesting that this phenomenon may have a direct role in membrane injury. In cases like RBC aging, pyruvate kinase deficient erythrocytes and irreversibly sickled cells, cation and water changes are related to adenosine tri phosphate (ATP) depletion [79]. Since Cr helps maintain the Na⁺, K⁺-ATPase activity, it can be given to avoid K⁺ loss.

**Fig 10:**
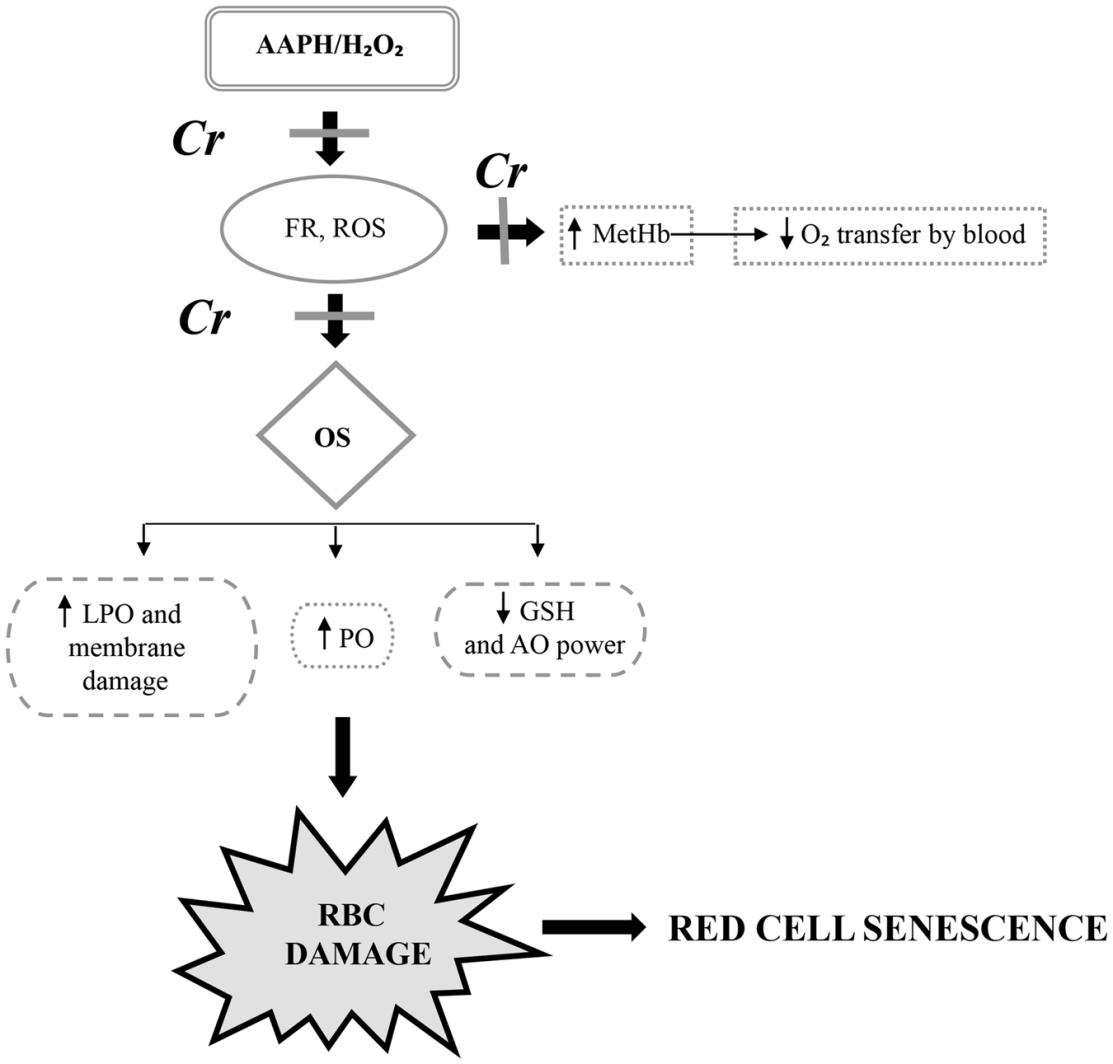
Schematic representation of H_2_O_2_/AAPH toxicity in human erythrocytes and protective effect of creatine. AAPH and H_2_O_2_ enter the cell and, being strong oxidants, increase the generation of free radicals and reactive oxygen species leading to oxidative stress condition. This converts hemoglobin to methemoglobin and decreases the oxygen carrying capacity of erythrocytes. Glutathione levels are lowered which reduces the antioxidant power of cell and results in protein oxidation, lipid peroxidation and membrane damage. All these factors contribute to cell damage which can reduce the lifespan of erythrocytes in blood (red cell senescence) since damaged erythrocytes are removed from circulation by the spleen. Creatine protects the erythrocytes either by inhibiting the generation of free radicals and reactive oxygen species or by directly quenching them by its antioxidant property. This protects the erythrocytes from oxidative damage and can enhance their lifespan. (GSH, glutathione; MetHb, methemoglobin; ROS, reactive oxygen species; FR, free radicals; PO, protein oxidation; LPO, lipid peroxidation; OS, oxidative stress; RBC, red blood cells).

In conclusion, our results clearly demonstrate that Cr can effectively protect human erythrocytes and lymphocytes from damage induced by oxidizing agents. It prevents protein oxidation, glutathione depletion, and conversion of hemoglobin to methemoglobin. Cr also shields lymphocyte DNA from oxidative injury and allows mitochondrial enzymes to stay active even in the presence of oxidant. This protection can be attributed to the intrinsic antioxidant properties of Cr. This is the first report that suggests a potential function for Cr in blood and also provides an explanation why lower Cr content results in red cell senescence. The protective effects of Cr on erythrocytes are summarized in Fig 10. Our results also indicate that creatinine, long considered an excretory end-product of Cr metabolism, may play a similar protective role in blood. However, further studies are necessary to delineate the exact molecular events underlying the mode of action of Cr (and also creatinine) to better exploit its therapeutic/preventive potential during supplementation.
